# Effect of Cold Saline Pre-Washing on Cement Leakage in Vertebroplasty: A Novel Approach

**DOI:** 10.3390/jcm14082755

**Published:** 2025-04-17

**Authors:** Réka Viola, Siran Aslan, Mohammad Walid Al-Smadi, András Gati, Konrád Szilágyi, Viktor Foglar, Árpád Viola

**Affiliations:** 1 Department of Psychiatry, Peterfy Sandor Hospital, 1076 Budapest, Hungary; pallareka1004@gmail.com; 2 Semmelweis Doctoral School, Semmelweis University, 1085 Budapest, Hungary; drsiran.5@gmail.com; 3 Neurotraumatology Division, Semmelweis University, 1081 Budapest, Hungary; arpadviola@gmail.com; 4 Department of Neurosurgery and Neurotraumatology, Dr. Manninger Jenő Traumatology Institute of Budapest, 1081 Budapest, Hungary; drgatiandras@gmail.com (A.G.); szilagyikonrad@gmail.com (K.S.); foglar.viktor@gmail.com (V.F.); 5 Faculty of Medicine, Semmelweis University, 1085 Budapest, Hungary

**Keywords:** percutaneous vertebroplasty, cement leakage, cold saline, polymethylmethacrylate, spinal fractures, osteoporotic vertebral compression fractures

## Abstract

**Background:** Cement leakage remains a significant challenge in percutaneous vertebroplasty (PVP). Leakage can lead to serious complications, including spinal cord compression, pulmonary embolism, and nerve root irritation. While several techniques have been proposed to minimize leakage, an effective and simple solution is still needed. This study investigates the impact of pre-washing vertebral bodies with cold saline before cement injection as a potential method to reduce leakage. **Methods:** A retrospective analysis was conducted on patients who underwent PVP for osteoporotic vertebral compression fractures. Patients were divided into three groups: (1) conventional PVP, (2) PVP with room-temperature saline pre-injection, and (3) PVP with cold saline (4 °C) pre-injection. Cement leakage was assessed using intraoperative fluoroscopy and postoperative computed tomography (CT), categorized into paravertebral, intervertebral, retrograde, spinal canal, and distant venous leakage. Statistical analysis was performed to compare leakage rates among the groups. **Results:** A total of 262 patients with 461 treated vertebrae were analyzed. Cold saline pre-treatment significantly reduced cement leakage rates compared to conventional PVP and room-temperature saline pre-injection (*p* < 0.05). CT imaging detected significantly more cement extravasation than fluoroscopy (*p* < 0.01). The incidence of spinal canal and intervertebral leakage was lowest in the cold saline group, suggesting improved cement containment and distribution. **Conclusions:** Pre-washing vertebral bodies with cold saline before cement injection in PVP significantly reduces cement leakage, particularly in the spinal canal and intervertebral spaces. This simple and cost-effective approach may enhance surgical safety and improve patient outcomes.

## 1. Introduction

Osteoporosis is a metabolic disorder that weakens bone structure, increasing fracture risk, particularly vertebral compression fractures (VCFs), which impair mobility, cause chronic pain, and elevate mortality [1,2]. The global burden is substantial, with osteoporosis causing 9 million fractures annually, including 1.4 million vertebral fractures, while in Europe, its impact surpasses most cancers [3].

Identifying osteoporotic vertebral fractures relies on advanced imaging, primarily CT and MRI. However, there is continued discussion about the most appropriate initial approach due to the greater time and resource demands of MRI. Nevertheless, MRI is especially effective in revealing subtle structural changes such as bone marrow edema, hemorrhage, and microfractures, which may not be visible on CT. While CT is more widely available, it can miss certain fractures, making MRI an essential tool for accurate diagnosis in unclear or early-stage cases [4,5,6,7].

Recent insights into the genetic and epigenetic mechanisms underlying osteoporotic vertebral fractures suggest that these factors significantly influence treatment response. While environmental contributors remain difficult to quantify, identifying molecular pathways and biomarkers may allow for a more accurate assessment of spinal fragility fracture burden across populations. Integrating multi-omics approaches with imaging advances holds promise for developing clinically relevant tools to improve diagnosis, patient stratification, and therapeutic outcomes [8].

The management of osteoporotic vertebral fractures can be either conservative or interventional. Non-invasive approaches typically include analgesia, rest, orthotic bracing, physiotherapy, and gradual rehabilitation. However, a subset of patients continues to experience persistent pain and functional impairment despite conservative treatment. For these patients, percutaneous vertebroplasty (PVP) has emerged as an effective minimally invasive procedure [9].

PVP restores vertebral stability by filling the fractured spaces and preventing micromotion between bone fragments. Additionally, the thermal and chemical properties of PMMA contribute to pain relief through the destruction of intraosseous nerve endings [9,10].

Despite its clinical benefits, PVP carries risks, with cement leakage being one of the most concerning complications. The extravasation of bone cement into surrounding structures—such as the spinal canal, nerve roots, and venous system—can result in neurological deficits, pulmonary embolism, or even fatal outcomes [11,12,13]. Various strategies have been proposed to minimize leakage, including precise needle placement, optimal cement viscosity, and adherence to manufacturer recommendations [14]. However, these measures do not eliminate the risk entirely.

We aimed to assess whether pre-injecting cold physiological saline (4 °C) into the vertebral body before cement augmentation reduces the risk of cement leakage. By evaluating its effect on cement setting and distribution, we seek to determine if this approach can enhance procedural safety compared to traditional PVP and PVP with room-temperature saline pre-injection.

## 2. Materials and Methods

### 2.1. Study Design and Population

This retrospective study analyzed 262 patients who underwent percutaneous vertebroplasty for thoracolumbar compression fractures between 1 July 2019 and 31 July 2021. A total of 461 vertebrae were treated. Patients were categorized into three groups based on the vertebroplasty technique used:

- Group I (classic PVP) consisted of 62 patients with 118 vertebrae who underwent conventional percutaneous vertebroplasty without saline pre-treatment.
- Group II (room-temperature saline PVP) included 158 patients with 175 vertebrae, where the vertebral body was washed three times using 20 mL of room-temperature saline before cement injection.
- Group III (cold saline PVP) involved 42 patients with 168 vertebrae, where the vertebral body was washed three times using 20 mL of 4 °C saline before cement injection.

The inclusion criteria were as follows: (1) thoracolumbar compression fractures classified as A1, A2, A3, or A4 according to the AO Spine Classification, (2) age of 50 years or older, (3) a T-score of −2.5 or lower for lumbar or femoral bone mineral density (BMD), (4) inadequate pain relief after four weeks of conservative treatment or severe pain, (5) osteoporotic vertebral compression fractures without posterior wall damage or nerve involvement, and (6) a preoperative visual analog scale (VAS) pain score of 7 or higher.

Exclusion criteria included the following: (1) pathological fractures caused by malignancy or infection, (2) previous spinal surgery at the treated level, (3) severe spinal deformities preventing safe percutaneous vertebroplasty access, (4) severe comorbidities affecting the heart, liver, kidney, or lungs, and (5) the presence of neurological deficits.

### 2.2. Surgical Procedure

All procedures were performed under fluoroscopy guidance. The affected vertebral level was identified using a C-arm X-ray, and a 2.5 mm trocar needle was introduced via a transpedicular approach on both sides of the vertebral body. In Groups II and III, the vertebral body was washed and filled before cement injection, specifically Polymethylmethacrylate (PMMA) injection. The procedure involved two 20 mL injections per side, with synchronization between filling one side of the vertebra with saline and withdrawing it from the other side, ensuring uniform distribution. A total of 60 mL of saline was injected per vertebra. In group II, room-temperature saline was used, while in group III, 4 °C saline was applied. Following saline preparation, polymethylmethacrylate bone cement was injected incrementally in 0.5 mL steps under real-time fluoroscopic monitoring. If cement leakage was observed, injection was immediately halted. After the procedure, all patients underwent anteroposterior and lateral X-rays, followed by a CT scan to assess cement distribution and complications.

The number of cement leakages at different anatomical locations, as identified on X-ray and CT, was recorded. Additionally, the amount of cement that could be injected before leakage occurred or, in the absence of leakage, the total amount injected, was documented for each side of the vertebral body. The relationship between the three surgical groups and the incidence of cement leakage was analyzed.

### 2.3. Radiographic Evaluation

The sensitivity of the X-ray and CT scans for the detection of cement leakages and the possibility of leakages in the intervertebral (discus), retrograde, paravertebral, vertebral canal, and distant venous sites were tested separately in the three surgical groups with the chi-square test of homogeneity and independence. The patient identifier, the diagnosis, the weight and height of the patients, the location of the treated vertebra(s), the surgeons’ and assistants’ names, the performed surgery, the date, start, and end of the procedure, and the blood loss were recorded. When augmented minimally invasive spine surgery was performed, then the size of the screw and the exact time during the surgery when cement was injected, a cage was inserted, or a dorsal stabilization was created were also noted.

### 2.4. Statistical Analysis

Data were statistically analyzed using GraphPad Prism 10 software (GraphPad Software, San Diego, CA, USA). We used descriptive statistics, including the mean ± standard deviation (SD), to summarize continuous variables and frequencies. The association between the cement leakages and the three surgical methods was examined using a chi-square test, with a *p*-value of less than 0.05 considered statistically significant.

## 3. Results

### 3.1. Cement Leakage Distribution

A total of 262 patients with 461 treated vertebrae were analyzed across the three study groups. The distribution of cement leakage was evaluated using intraoperative and postoperative X-rays and confirmed via CT scans, which demonstrated significantly higher sensitivity in detecting leakages (*p* < 0.01) (Table 1).

In group I (classic PVP, mean age: 68.7 ± 9.7 years), the intra- and postoperative X-ray detected 21 paravertebral venous, 13 paravertebral diffuse, 34 spinal canal, 42 intervertebral, 9 distant, and 11 retrograde leakages. The postoperative CT scan identified a higher number of leakages, detecting 86 paravertebral venous (13 anterior and 73 lateral), 21 paravertebral diffuse (10 anterior and 11 lateral), 45 spinal canal, 44 intervertebral, 22 distant, and 22 retrograde leakages.

In group II (room-temperature saline PVP, mean age: 69.2 ± 8.9 years), the intra- and postoperative X-ray detected 91 paravertebral venous, 8 paravertebral diffuse, 76 spinal canal, 20 intervertebral, 14 distant, and 7 retrograde leakages. The postoperative CT scan revealed 146 paravertebral venous (12 anterior and 134 lateral), 15 paravertebral diffuse (4 anterior and 11 lateral), 103 spinal canal, 32 intervertebral, 18 distant, and 17 retrograde leakages.

In group III (cold saline PVP, mean age: 69.7 ± 8.3 years), the intra- and postoperative X-ray detected 76 paravertebral venous, 3 paravertebral diffuse, 25 spinal canal, 4 intervertebral, 21 distant, and 11 retrograde leakages. The postoperative CT scan detected 145 paravertebral venous (13 anterior and 132 lateral), 13 paravertebral diffuse (1 anterior and 12 lateral), 68 spinal canal, 4 intervertebral, 28 distant, and 11 retrograde leakages.

### 3.2. Comparison of Cement Leakage Between Groups

A statistical comparison was performed to analyze leakage incidence across the three groups. Paravertebral leakage rates did not differ significantly among the groups (*p* > 0.05). However, significant differences were found in spinal canal, intervertebral, and retrograde leakages (Table 2).

Spinal canal leakage was significantly lower in group III compared to group II (*p* < 0.05). No significant difference was observed between groups I and II. Intervertebral leakage was significantly lower in group II than in group I (*p* < 0.01), with group III demonstrating an even further reduction (*p* < 0.01). Retrograde leakage was significantly lower in group II than in group I (*p* < 0.05), with group III exhibiting the lowest leakage rate overall (*p* < 0.01). No significant difference was found in distant venous leakage across groups (*p* > 0.05). These findings confirm that the cold saline pre-treatment significantly reduces spinal canal, intervertebral, and retrograde leakages compared to the traditional PVP and room-temperature saline pre-treatments.

## 4. Discussion

Vertebral fractures significantly impact patients’ quality of life, leading to chronic pain, reduced mobility, and increased morbidity. Percutaneous vertebroplasty is widely performed for pain relief and stabilization, yet cement leakage remains a major concern.

The literature suggests several techniques to minimize complications, including selecting a transpedicular approach for the lumbar spine and the costovertebral junction for the thoracic spine, avoiding cortical breaches whenever possible, optimizing cement opacification by adhering to manufacturer guidelines, maintaining the recommended powder-to-liquid polymer ratio, and ensuring proper viscosity before injection [13,14].

Despite these precautions, leakage rates are still ranging from 34 to 91.3 percent, according to Noguchi et al. Leakage can result in neurological deficits, pulmonary embolism, and severe complications, necessitating improved techniques to reduce risk [9].

To further reduce cement leakage, several novel techniques have been explored. O-arm navigation enhances puncture accuracy, lowering the needle malposition rate to 4.5 percent and enabling real-time visualization of cement distribution, unlike conventional C-arm-guided vertebroplasty. This allows the immediate detection and management of potential leakage during the procedure. Additionally, O-arm imaging has proven highly effective in guiding biopsy and ablation procedures across various spinal pathologies, suggesting its potential value in complex vertebroplasty cases requiring precise needle placement [15,16,17,18]. This allows the immediate detection and management of potential leakage during the procedure. Another approach is the unilateral extrapedicular technique, which minimizes the risk of cement migration into the spinal canal while ensuring adequate bilateral cement distribution [19].

In this study, we assessed the effect of pre-injecting cold saline into the vertebral body before cement augmentation. Our results demonstrate that cold saline pre-treatment significantly reduces the incidence of cement leakage into the spinal canal, intervertebral space, and retrograde locations compared to classic percutaneous vertebroplasty and percutaneous vertebroplasty with room-temperature saline pre-injection. This suggests that pre-washing with cold saline improves cement retention within the vertebra, thereby reducing extravasation into adjacent structures.

Bone cement is a dual-component system comprising a liquid and a powder component. In the powder phase, in addition to methylmethacrylate copolymers, benzoyl peroxide (BPO) and zirconium or barium are also present, which are utilized to provide radio-opacity [20]. The liquid mainly contains monomers. When the two components are mixed, polymerization is initiated.

Polymethylmethacrylate is a self-curing acrylic polymer used extensively in vertebroplasty due to its mechanical strength, rapid setting time, and ability to stabilize fractures. It is composed of a liquid monomer and a powder polymer, which undergo an exothermic polymerization reaction upon mixing. This reaction generates heat, reaching temperatures as high as 77.3 degrees Celsius at the core, facilitating polymer chain formation and hardening. However, excessive heat production can damage surrounding tissues and alter cement behavior, making leakage more likely [21,22].

Cooling polymethylmethacrylate with cold saline pre-injection may improve its handling properties by influencing its viscosity and polymerization kinetics. Lower temperatures slow the exothermic reaction, allowing the cement to achieve a thicker, more stable consistency earlier. This increased viscosity prevents excessive spread, reducing the risk of leakage into unintended spaces.

Additionally, cold saline enhances spherical droplet formation, ensuring more localized and controlled cement distribution within the vertebra, which may explain the reduced leakage rates observed in our study. This hypothesis is consistent with findings from Zhu et al., who emphasized the role of polymerization kinetics in the behavior of PMMA during augmentation procedures [23]. Moreover, Szoradi et al. have shown that polymerization-induced thermal effects at the bone–cement interface can influence tissue integrity, further supporting the premise that pre-cooling may enhance safety and visualization [24].

Our results align with and expand upon previous research aimed at reducing cement leakage in vertebroplasty. Hulme et al. reported a cement leakage rate of 72 percent in their vertebroplasty cohort, with the most frequent locations being paravertebral veins and intervertebral disc spaces. Our study found similar overall leakage rates in the classic percutaneous vertebroplasty group, but the groups treated with room-temperature and cold saline exhibited significantly reduced intervertebral and spinal canal leakage rates, particularly when cold saline was used [25].

Kan et al. reported that both high-viscosity and low-viscosity bone cement in percutaneous vertebroplasty provide similar pain relief; however, high-viscosity cement offers procedural advantages by shortening operative time, reducing fluoroscopy exposure, minimizing vertebral cement leakage, and improving surgical safety [26]. In contrast, Alhashash et al., found that cement viscosity does not significantly influence clinical outcomes, but lower viscosity and severe osteoporosis are key risk factors for cement leakage. Additionally, they identified that the number of vertebral fractures at presentation predicts new fractures postoperatively [27].

Another study by Xiao et al. examined the effect of polymethylmethacrylate temperature on leakage rates, finding that pre-cooled cement that was stored overnight in a refrigerator at four degrees Celsius significantly reduced extravasation [28]. This is consistent with our results, suggesting that temperature plays a key role in cement handling. However, our study differs in that cold saline is injected into the vertebral body rather than cooling the polymethylmethacrylate itself, allowing for better interaction with bone architecture before cement injection.

### Strength and Limitations of the Study

The use of cold saline before polymethylmethacrylate injection offers a novel, practical approach to reducing cement leakage risk in vertebroplasty. This technique could be readily incorporated into standard clinical practice given its simplicity and effectiveness. Our results suggest that this method is particularly beneficial in reducing spinal canal and intervertebral leakages, which are among the most clinically significant complications.

This study has some limitations. First, its retrospective nature may introduce selection bias. Second, while our findings demonstrate a significant reduction in cement leakage, we did not directly measure changes in polymethylmethacrylate polymerization kinetics, which would require laboratory-based experiments. Finally, patient follow-up data on long-term functional outcomes and cement integrity over time were not included and should be considered in future research. Future studies should include multicenter and prospective cohorts to validate these preliminary findings and explore the mechanistic basis of cold-saline-induced thermal modulation during vertebroplasty.

## 5. Conclusions

Pre-injecting cold saline before cement augmentation significantly reduces cement leakage into the spinal canal, intervertebral space, and retrograde locations in vertebroplasty. The cooling effect likely enhances cement viscosity control, reduces spread, and improves distribution within the vertebra. Given its simplicity and effectiveness, this approach could be a valuable addition to vertebroplasty protocols, improving patient safety and reducing procedure-related complications.

## Figures and Tables

**Table 1:** Cement leakage distribution across groups. PVP: percutaneous vertebroplasty.

Group	Paravertebral (Venous)	Paravertebral (Diffuse)	Spinal Canal	Intervertebral	Retrograde	Distant	Total Cement Leakages
X-ray Detection							482
Group I (Classic PVP)	21	13	34	42	11	9	130
Group II (RT Saline PVP)	91	8	76	20	7	14	216
Group III (Cold Saline PVP)	76	3	25	4	11	21	136
CT Detection							840
Group I (Classic PVP)	86 (13 Anterior and 73 Lateral)	21 (10 Anterior and 11 Lateral)	45	44	22	22	240
Group II (RT Saline PVP)	146 (12 Anterior and 134 Lateral)	15 (4 Anterior and 11 Lateral)	103	32	17	18	331

**Table 2:** Statistical comparison of cement leakage across groups; * reflects statistical significance.

Group Comparison	Paravertebral	Distant	Retrograde	Intervertebral	Spinal Canal
Group I vs. II	*p* > 0.05	*p* > 0.05	*p* < 0.05 *	*p* < 0.01 *	*p* > 0.05
Group II vs. III	*p* > 0.05	*p* > 0.05	*p* < 0.01 *	*p* < 0.01 *	*p* < 0.05 *
Group I vs. III	*p* > 0.05	*p* > 0.05	*p* < 0.01 *	*p* < 0.01 *	*p* < 0.05 *

## Data Availability

All data generated or analyzed during this study are included in this published article.
